# Defining the Optimal Method for Measuring Metabolic Tumor Volume on Preoperative ^18^F-Fluorodeoxyglucose-Positron Emission Tomography/Computed Tomography as a Prognostic Predictor in Patients With Pancreatic Ductal Adenocarcinoma

**DOI:** 10.3389/fonc.2021.646141

**Published:** 2021-03-12

**Authors:** Yasuko Tatewaki, Chiaki Maeda Terao, Kyohei Ariake, Ryoko Saito, Tatsushi Mutoh, Hideo Shimomura, Fuyuhiko Motoi, Masamichi Mizuma, Hayato Odagiri, Michiaki Unno, Yasuyuki Taki

**Affiliations:** ^1^ Department of Nuclear Medicine and Radiology, Institute of Development, Aging and Cancer, Tohoku University, Sendai, Japan; ^2^ Department of Geriatric Medicine and Neuroimaging, Tohoku University Hospital, Sendai, Japan; ^3^ Department of Surgery, Tohoku University Graduate School of Medicine, Sendai, Japan; ^4^ Department of Pathology, Tohoku University Graduate School of Medicine, Sendai, Japan; ^5^ Kousei Sendai Clinic, Sendai, Japan; ^6^ Department of Surgery I, Yamagata University Graduate School of Medical Science, Yamagata, Japan; ^7^ Division of Radiology, Tohoku University Hospital, Sendai, Japan; ^8^ Smart-Aging International Research Center, Tohoku University, Sendai, Japan

**Keywords:** prognosis, metabolic tumor volume (MTV), threshold, FDG PET = F-18 fluorodeoxyglucose positron emission tomography, PDAC=pancreatic ductal adenocarcinoma

## Abstract

**Objectives:**

Metabolic tumor volume (MTV) on ^18^F-fluorodeoxyglucose-positron emission tomography/computed tomography (FDG-PET/CT) is a promising prognostic predictor in pancreatic ductal adenocarcinoma (PDAC). However, the optimal segmentation method and threshold value to determine MTV for PDAC are still unclear. We explored the optimal method and threshold value for the prognostic value of MTV measured on pre-treatment ^18^F-FDG-PET/CT.

**Methods:**

Seventy-three patients with resected PDAC who underwent ^18^F FDG-PET/CT before surgical resection were enrolled. MTV values of the tumor were measured on FDG-PET/CT by the two fixed-threshold methods using threshold values as 2.0, 2.5, 3.0, and 3.5 for the absolute method and 35%, 40%, 42%, 45%, and 50% for the relative method. Receiver operating characteristic curve analysis for prediction of 1-year survival rates was conducted for determining the optimal threshold values, and we selected the optimal method and threshold value considering area under the curve. The prognostic values of each FDG-PET/CT parameter for disease-specific survival and recurrence-free survival were assessed with Kaplan–Meier method and Cox proportional hazard models.

**Results:**

In receiver operating characteristic curve analysis, MTV by the fixed-absolute threshold method based on a threshold value of 3.5 (MTV3.5) performed best in our study with area under the curve 0.724, sensitivity of 65%, and specificity of 75%. In univariate and multivariate analyses, MTV3.5 was significantly associated with disease-specific and recurrence-free survival.

**Conclusions:**

MTV3.5 by absolute threshold on pre-treatment FDG-PET/CT was the best independent prognostic predictor in resectable PDAC compared with other absolute threshold values and relative threshold values.

## Introduction

Pancreatic ductal adenocarcinoma (PDAC) is one of the most lethal carcinomas and the fourth leading cause of cancer-related death in Japan (1). Although surgical resection is the only potentially curative treatment for PDAC, the prognosis after resection remains poor because of the high incidence of recurrence (2, 3). Studies have demonstrated the ability of ^18^F-fluorodeoxyglucose positron emission tomography/computed tomography (^18^F-FDG PET/CT) to assess PDAC because of its delineation of tumor glucose metabolic activity as well as tumor burden (4). High FDG uptake indicates malignant properties in tumors. Additional information regarding pre-surgical tumor metabolism could contribute to the selection of more effective treatment strategies, such as operation, systemic chemotherapy, and radiation therapy, alone or in combination.

The most widely used parameter of ^18^F-FDG PET/CT is the maximum standardized uptake value (SUVmax) because it is easily measured as a semiquantitative parameter, and it shows high reproducibility. In a previous study, we found that SUVmax is a useful parameter for predicting the prognosis of patients with pancreatic cancer (5). However, SUVmax is just a single-pixel value within a region of interest, and SUVmax is subject to considerable noise (6–8). Therefore, SUVmax is unlikely to accurately reflect the tumor metabolic activity, especially in heterogenous tumors. Recently, the volumetric parameters associated with ^18^F-FDG PET/CT such as metabolic tumor volume (MTV) and total lesion glycolysis (TLG) were reported to be effective prognostic factors in patients with PDAC (9–14). These parameters have been considered more reliable for predicting prognosis than SUVmax because they reflect not only tumor glucose metabolism level but also whole tumor burden (15).

Numerous segmentation methods for MTV have been developed, including threshold-based methods and algorithm-based methods, and the MTV values are significantly affected by the segmentation method (16, 17). The algorithm method, including the gradient-based method, is more advanced and sophisticated than threshold-based methods, and this method requires familiarity with professional image analysis software. In contrast, threshold-based segmentation methods can be easily implemented and widely used because of their simplified methodology. Clinically, MTV is often determined using an approach based on two fixed-threshold methods: fixed-absolute threshold method and fixed-relative threshold method of the SUVmax in the tumor (18). Regarding absolute SUV thresholds, all voxels with SUV above the fixed-absolute threshold value within the VOI are assigned to the tumor and those below the threshold are considered background. SUV2.5 is the most widely accepted threshold because of its consistently good prediction for prognosis (9). For relative thresholds, which are defined as a certain percentage of the SUVmax of a tumor, 40% or 42% are the most widely accepted thresholds for their predictive values (19). The optimal segmentation method to measure MTV has varied depending on the characteristics of the tumors or the purpose of the study; however, confirming a standardized method for PDAC has been controversial. To the best of our knowledge, most of the previous studies that investigated the prognostic value of MTV and TLG in PDAC patients only used one method to determine the MTV (10, 11, 13, 14, 18). The exact method and precise threshold value that are associated with prognosis have not been confirmed.

We investigated the optimal fixed-threshold method of SUV and threshold values for determining volumetric parameters, including MTV and TLG, on pre-therapeutic FDG-PET/CT for predicting the prognosis of PDAC patients. The aim of this study was to establish the optimal methodology for measuring FDG-PET/CT parameters with PDAC and to confirm the clinical significance of these parameters.

## Materials and Methods

### Patient and Data Collection

This retrospective study enrolled 73 consecutive patients with PDAC at resectable stage (n=49) and borderline resectable stage (n=24) (20) who underwent curative resection for PDAC in the department of Surgery at Tohoku University Graduate School of Medicine (Sendai, Japan) between January 1, 2006 and December 31, 2016. All data and information were reviewed from medical records, operative reports, and pathological reports. Eligible patients had undergone ^18^F-FDG PET/CT at preoperative investigation. The exclusion criteria were as follows: 1) distant metastases at the time of initial surgery, 2) neoadjuvant chemotherapy (NAC), 3) died of comorbidities during their hospital stay, 4) low FDG-uptake of the tumor (SUVmax < 2.5), or 5) marked pancreatitis that could not be distinguished from tumor uptake on ^18^F-FDG PET/CT.

Postoperative follow-up was conducted at least once every 2–3 months until the patient’s death or the last day of data collection (March 31, 2020). Follow-up included blood tests including cancer antigen 19-9 (CA19-9), ultrasonography, magnetic resonance imaging, CT, and ^18^F-FDG PET/CT to detect cancer recurrence. Adjuvant chemotherapy was performed according to the standard protocols of our institution (5). This study was approved by the Institutional Review Board of Tohoku University (2016-1-573). The requirement of informed consent was waived, and an opt-out method was used because of the retrospective design of the study. The research was conducted in accordance with the Declaration of Helsinki.

### ^18^F-Fluorodeoxyglucose-Positron Emission Tomography Positron-Emission Tomography/CT Imaging Protocol

The ^18^F-FDG PET/CT examinations were performed using a Biograph Duo or Biograph 40 PET/CT system (Siemens Healthcare, Erlangen, Germany) from the skull base to the proximal thigh in a supine position. The patients were required to fast for a minimum of 4 h before the ^18^F-FDG injection. After injection of approximately 185.0–370.0 MBq of ^18^F-FDG, the patients rested for about 1 h before imaging. PET acquisition time was 2 min/bed position in three-dimensional mode, and images were reconstructed using the ordered subsets expectation maximization algorithm (14 subsets, 6 iterations) and point spread function correction model. The low-dose CT transmission scan was acquired with 140 kVp and 25 mAs and 2 mm slice thickness. PET images were displayed in a 168 × 168 matrix (pixel size 4.07 × 4.07 mm, slice thickness 2.0 mm). The reconstructed PET/CT images were reviewed by nuclear medicine radiologists (YTat, HS, and TM).

### Measurement of ^18^F-Fluorodeoxyglucose-Positron Emission Tomography Positron-Emission Tomography/CT Parameters

**Figure 1:**
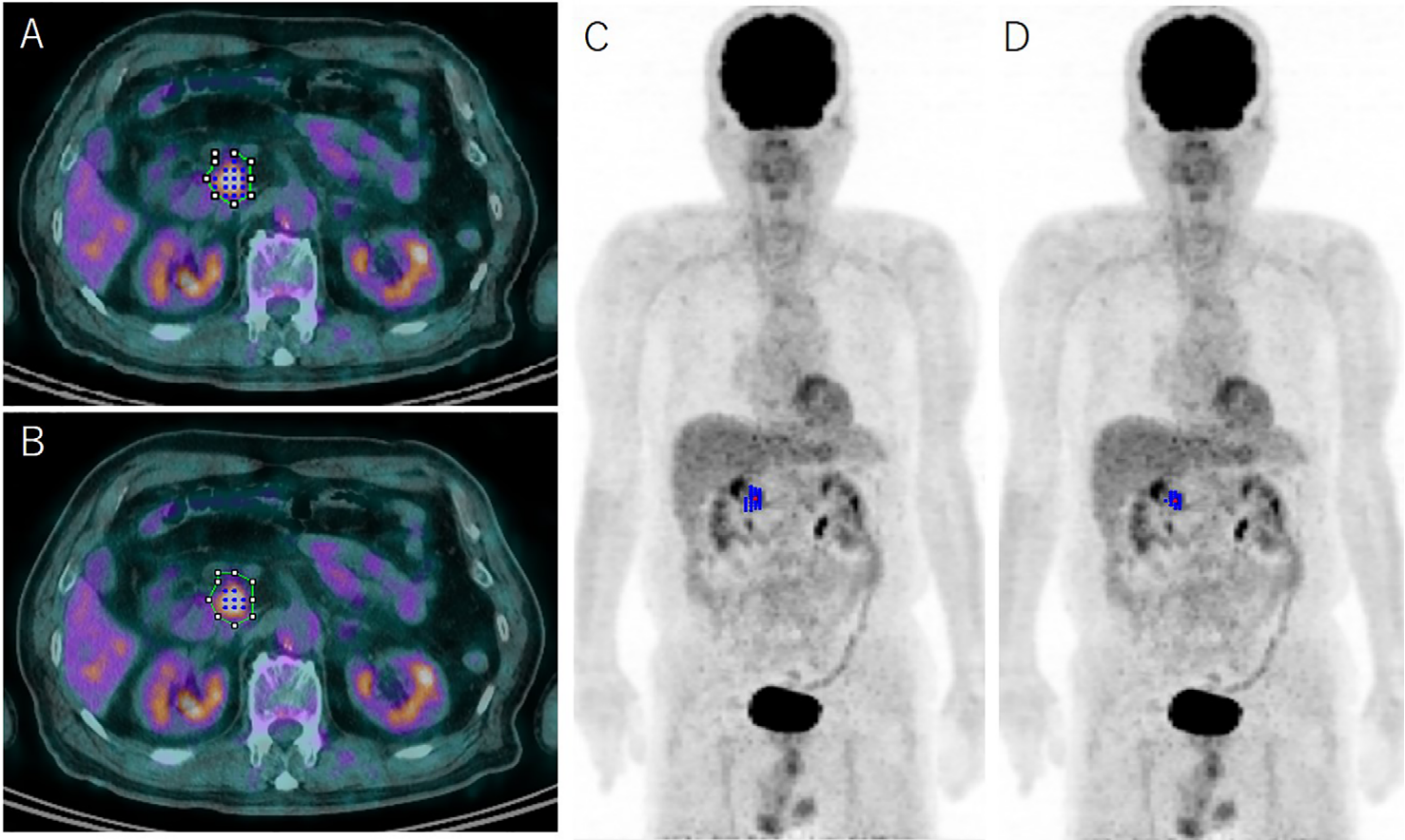
Representative case determined using the absolute 2.5 threshold method **(A, C)** and the relative 41% threshold method **(B, D)** with representative axial ^18^F-fluorodeoxyglucose-positron emission tomography (FDG-PET)/computed tomography fusion images and 3D reconstructed FDG-PET images. The green line on the axial images **(A, B)** indicates the initial volume of interest for determining metabolic parameters of pancreas ductal adenocarcinoma on FDG-PET; the blue dots and lines show the identical metabolic tumor volume (MTV) using the absolute 2.5 method **(A, C)** and the relative 41% method **(B, D)**, respectively. The MTV is grossly underestimated using the relative 41% method compared with the 2.5 method.

Using the reconstructed PET/CT images, the radiologists (YTat, CT, and HS) measured SUVmax, SUVmean, MTV, and TLG using the Beth-Israel PET-CT viewer plug-in (http://petctviewer.org) for ImageJ software available from FIJI (http://www.fiji.sc). The volume of interest (VOI) for estimating MTV was drawn around each focus of ^18^F-FDG uptake on pretreatment FDG-PET/CT (**Figure 1**). In each VOI, we measured PET parameters with various threshold values for the two methods for determining MTV: SUV of 2.0, 2.5, 3.0 and 3.5 for the fixed-absolute threshold method, selecting voxels above the thresholds, and 35%, 40%, 42%, 45% and 50% against SUVmax for the fixed-relative threshold method. We did not use fixed-threshold values less than SUV of 2.0 or less than 35% against the SUVmax in the VOIs because threshold values that are too low can lead to difficulty in discriminating the FDG uptake of the PDAC from that of background pancreas tissue. Conversely, setting the threshold values higher than SUV of 3.5 or more than 50% against the SUVmax in the VOIs could lead to MTV values that are too small to evaluate. TLG was calculated as (SUVmean) × (MTV) in each method.

### Statistical Analysis

1. Statistical analyses were conducted using JMP Pro software, version 14.0 (SAS Institute Inc., Cary, NC, USA). Data are expressed as the mean ± standard deviation or median and range, unless otherwise indicated.
2. For determining the optimal threshold values for the two methods for defining MTV, we conducted receiver operating characteristic (ROC) curve analysis for prediction of 1-year disease-specific survival (DSS) rates. SUVmax was a predictor of one-year DSS with high sensitivity in our previous study (Ariake et al., 2018). By determining values of area under the curve (AUC) for each ROC curve, we selected the optimal threshold value for the following analysis.
3. To determine the correlations between FDG-PET/CT parameters of PDAC and the clinicopathological data, linear regression analysis was conducted using Pearson or Spearman correlation coefficients, Chi-square test, and Wilcoxon-rank test. *P* < 0.05 was considered statistically significant.
4. Prior to survival analysis, all variables were grouped into two categories according to the cutoff value. ROC curve analysis was used to determine the optimal cutoff values for prediction of 1-year survival rates. Survival rates were established using the Kaplan–Meier method and the differences in survival between the groups were compared using log-rank tests. DSS was measured from the date of surgical resection until the date of death or censoring. Recurrence-free survival (RFS) times were measured from the date of surgical resection until the date of recurrence or censoring. For multivariate analysis, independent prognostic factors were identified using a Cox proportional hazards regression model. *P* < 0.05 was considered statistically significant.

## Results

### Patient Characteristics

Of the 73 patients, 55 patients (75.3%) showed recurrence and 53 patients (72.6%) died during the follow-up period. The median duration of clinical follow-up was 30.3 months (range: 2.7–116.8 months). The demographics and patient characteristics are shown in **Table 1**. The initial postoperative recurrence sites were locoregional sites (n=29), liver (n=28), and peritoneum (n=12); some patients had multiple recurrences.

**Table 1:** Patient and tumor characteristics (n = 73).

Characteristic	N
Sex: male/female	47/26
Age, years, mean ± SD (range)	69.2 ± 9.4 (48–85)
Resectability: R/BR	49/24
Tumor location: head/body and tail	47/26
Operation type: PD/TP/DP	41/8/24
Adjuvant treatment: yes/no	58/14
Tumor size, mm, mean ± SD (range)	29.1 ± 10.1 (15–65)
Residual cancer: R0/R1	67/6
pT: 2/3/4	2/44/27
Lymph node metastasis: yes/no	47/26
Differentiation: well/mod/poor	13/53/7
Pretreatment CA19-9, U/ml, mean ± SD (range)	535.9 ± 1302.0 (1.5–8901)
SUVmax, mean ± SD (range)	5.1 ± 1.8 (2.6–9.4)
MTV3.5, mean ± SD (range)	3.78 ± 5.43 (0–29.64)
TLG 3.5, mean ± SD (range)	16.67 ± 23.07 (0–104.7)

BR, borderline resectable; CA19-9, cancer antigen 19-9; DP, distal pancreatectomy; MTV, mean tumor volume; PD, pancreaticoduodenectomy; R, resectable; SD, standard deviation; SUVmax, maximum standardized uptake value; TLG, total lesion glycolysis; TP, total pancreatectomy.

### Optimal Fixed-Threshold Method and Value for MTV

**Figure 2:**
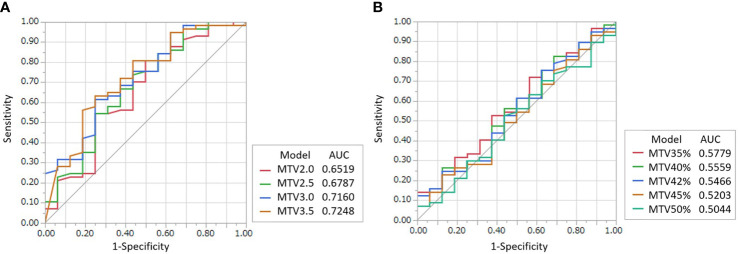
ROC curves for determining the optimal threshold value of the fixed-absolute threshold method of MTV **(A)** and that of the fixed-relative threshold method of MTV **(B)** for predicting 1-year DSS. The threshold values for the fixed-absolute threshold method were 2.0, 2.5, 3.0 and 3.5. The threshold values for the fixed-relative threshold method were 35%, 40%, 42%, 45% and 50%. The tables on the right of the graphs show area under the curve (AUC) with 95% confidence intervals. The MTV using the 3.5 absolute-threshold value was the best predictor for DSS (AUC: 0.7248).

The optimal fixed-threshold method and values for predicting prognosis were determined using ROC curve analysis. The AUCs and cutoff values of MTV and TLG from the nine fixed-threshold values (SUV of 2.0, 2.5, 3.0, and 3.5 for the fixed-absolute threshold method and 35%, 40%, 42%, 45%, and 50% against SUVmax for the fixed-relative threshold method) for 1-year DSS outcome are shown in **Figure 2** and **Supplementary Table 1**. The cutoff value of 2.46 on a fixed-absolute threshold value of 3.5 (MTV3.5) was the most optimal cutoff value with a sensitivity of 65% and a specificity of 75%. Therefore, we selected this value in this study.

### Correlations Between MTV3.5 and TLG3.5 Based on Fluorodeoxyglucose-Positron Emission Tomography Positron-Emission Tomography/CT and Clinicopathologic Parameters

The correlation analysis results of MTV3.5 and TLG3.5 based on FDG-PET/CT and clinicopathologic parameters are shown in **Table 2**. SUVmax, MTV3.5, and TLG3.5 showed correlations with resectability. MTV3.5 and TLG3.5 showed a strong positive correlation with tumor size. Only SUVmax had statistically significant correlations with lymph node metastasis.

**Table 2:** Association of FDG-PET/CT parameters with clinicopathological parameters.

	SUVmax			MTV3.5			TLG3.5		
	mean ± SD	R^2^	P	mean ± SD	R^2^	P	mean ± SD	R^2^	P
Sex			0.115			0.161			0.141
Male	4.75 ± 1.60			3.11 ± 4.68			13.71 ± 20.98		
Female	5.49 ± 2.06			4.98 ± 6.50			22.03 ± 26.00		
Age		0.03	0.135		<0.001	0.808		<0.001	0.985
Pre-treatment CA19-9		0.011	0.380		0.039	0.093		0.048	0.063
Resectability			0.0379*			0.0125^*^			0.0096^†^
R	4.74 ± 1.59			2.68 ± 3.67			11.84 ± 16.53		
BR	5.66 ± 2.04			6.02 ± 7.51			26.53 ± 30.74		
Tumor size		0.051	0.053	0.2562		<0.0001^§^	0.273		<0.0001^§^
Differentiation			0.333			0.7130			0.6944
Well	4.43 ± 1.54			4.29 ± 4.97			16.45 ± 29.40		
Mod	5.24 ± 1.83			4.01 ± 4.97			18.25 ± 22.81		
Poor	4.83 ± 1.97			1.88 ± 2.25			8.62 ± 11.75		
pT			0.077			0.1820			0.1102
2	4.07 ± 1.99			2.15 ± 3.03			9.06 ± 12.81		
3	4.72 ± 1.70			2.91 ± 5.28			12.51 ± 20.94		
4	5.64 ± 1.80			5.30 ± 5.60			24.03 ± 25.53		
LN metastasis			0.0437*			0.6441			0.4716
Yes	5.61 ± 1.89			3.55 ± 6.00			15.21 ± 24.46		
No	4.73 ± 1.67			4.17 ± 4.31			19.31 ± 20.50		

Data are expressed as mean ± SD (range) or number of patients.

*P < 0.05; ^†^P < 0.01; ^§^P < 0.001.

BR, borderline resectable; CA19-9, cancer antigen 19-9; LN, lymph node; MTV, mean tumor volume; R, resectable; SD, standard deviation; SUVmax, maximum standardized uptake value; TLG, total lesion glycolysis.

Characteristics of MTV2.5 and MTV41%, which were predominant threshold values for each fixed-absolute and fixed-relative threshold method (10, 11, 13, 14, 18), against tumor size and SUVmax are shown in **Supplementary Figure 1**.

### Evaluation of Prognostic Factors of MTV3.5 and TLG3.5 for Disease-Specific Survival and Recurrence-Free Survival

Age, sex, pretreatment CA19-9, resectability (resectable or borderline resectable), SUVmax, MTV3.5, and TLG3.5 were evaluated for DSS and RFS because they are clinically important variables that are available throughout pre-surgical examinations. ROC curve analysis of 1-year DSS revealed predictive cutoff values of 66 years for age (AUC 0.596), 246.4 U/mL for pre-treatment CA19-9 levels (AUC 0.641), and 4.87 for SUVmax on FDG/PET-CT (AUC, 0.713).

**Figure 3:**
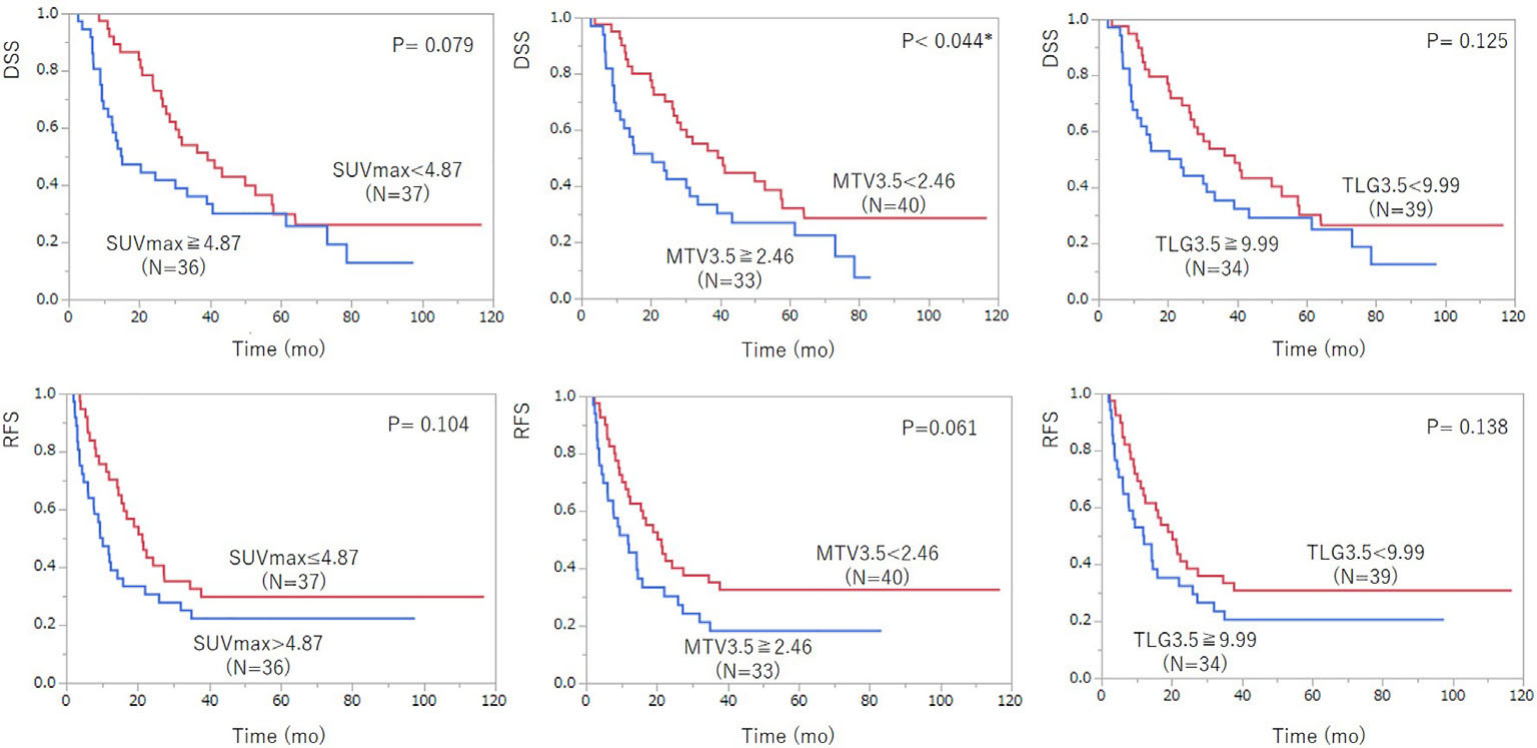
Kaplan–Meier survival analysis of ^18^F-FDG PET/CT parameters including SUVmax, MTV3.5, and TLG3.5 according to disease-specific survival (DSS) (top row) and recurrent-free survival (RFS) after pancreatic cancer surgery (bottom row). Among the ^18^F-FDG PET/CT parameters, MTV3.5 showed statistically significant prediction ability for DSS and had a tendency for RFS. TLG3.5 was not a significant prognostic indicator for DSS and RFS.

In univariate analysis, age, pre-treatment serum CA19-9 level, and MTV3.5 were significantly associated with DSS. SUVmax and resectability were not significant but showed some tendency in prediction of DSS (SUVmax: *P* = 0.079; resectability: *P* = 0.075) (**Table 3**, **Figure 3**). For predicting RFS, only pre-treatment serum CA19-9 level was statistically significant, but MTV3.5 showed a tendency in prediction of RFS (*P* = 0.061). In multivariate analysis, SUVmax and MTV3.5 were significant for predicting both DSS and RFS, but TLG3.5 was not significant (**Table 4**, **Table 5**).

**Table 3:** Prognostic factors in univariate analysis.

	Number of patients	DSS		RFS	
		Median (mo)	P	Median (mo)	P
Sex			0.230		0.5576
Male	47	26.2		14.3	
Female	26	40.2		18.7	
Age			0.0343*		0.1504
<66	24	40.7		15.9	
≥66	49	27.1		15.0	
Pre-treatment serum CA19-9 (U/ml)			0.0195*		0.0148*
<276.4	47	40.0		19.6	
≥276.5	26	23.8		8.3	
Resectability			0.0748		0.2302
R	49	39.9		21.3	
BR	24	14.4		10.7	
SUVmax			0.0791		0.1041
<4.87	37	39.3		21.3	
≥4.87	36	15.0		9.9	
MTV3.5			0.0444*		0.0608
<2.46	48	40.1		20.8	
≥2.46	25	20.4		11.9	
TLG3.5			0.1253		0.138
<9.99	38	39.3		20.2	
≥9.99	35	22.1		12.0	

Data are expressed as median ± SD (range) or number of subjects.

*P < 0.05.

BR, borderline resectable; CA19-9, cancer antigen 19-9; DSS, disease-specific survival; MTV, mean tumor volume; R, resectable; RFS, recurrence-free survival; SD, standard deviation; SUVmax, maximum standardized uptake value; TLG, total lesion glycolysis.

**Table 4:** Multivariate analysis with SUVmax, MTV3.5, and TLG3.5 for DSS.

	DSS model with SUVmax			DSS model with MTV3.5			DSS model with TLG3.5		
	P	Hazard ratio	95% CI	P	Hazard ratio	95% CI	P	Hazard ratio	95% CI
Sex	0.045*	1.822	1.013–3.277	0.031*	1.923	1.062–3.481	0.066	1.723	0.965–3.079
Age ≥66	0.050	1.928	1.000–3.715	0.066	1.845	0.066–0.961	0.075	1.813	0.942–3.492
Resectability: BR	0.011*	2.199	1.198–4.38	0.039*	1.844	1.033–3.292	0.031*	1.895	1.061–3.384
CA19-9 ≥276.5	0.140	1.541	0.867–2.739	0.082	1.665	0.938–2.956	0.139	1.542	0.868–2.737
SUVmax ≥4.87	0.016*	2.018	1.142–3.565						
MTV3.5 ≥2.46				0.011*	2.076	1.187–3.632			
TLG3.5 ≥9.99							0.089	1.601	0.930–2.771

BR, borderline resectable; CA19-9, cancer antigen 19-9; CI, confidence interval; DSS, disease-specific survival; MTV, mean tumor volume; RFS, recurrence-free survival; SD, standard deviation; SUVmax, maximum standardized uptake value; TLG, total lesion glycolysis.

*P < 0.05.

**Table 5:** Multivariate analysis with SUVmax, MTV3.5, and TLG3.5 for RFS.

	RFS model with SUVmax			RFS model with MTV3.5			RFS model with TLG3.5		
	P	Hazard ratio	95% CI	P	Hazard ratio	95% CI	P	Hazard ratio	95% CI
Sex	0.267	1.386	0.778–2.467	0.230	1.425	0.800–2.539	0.331	1.327	0.750–2.349
Age ≥66	0.282	1.421	0.749–2.695	0.366	1.340	0.711–2.526	0.380	1.329	0.704–2.510
CA19-9 ≥276.5	0.058	1.748	0.982–3.112	0.040*	1.829	1.030–3.250	0.060	1.936	0.978–3.084
Resectability, BR	0.098	1.651	0.912–2.988	0.219	1.435	0.807–2.553	0.187	1.473	0.187–0.828
SUVmax ≥4.87	0.041*	1.797	1.026–3.149						
MTV3.5 ≥2.46				0.030*	1.849	1.062–3.220			
TLG3.5 ≥9.99							0.128	1.527	0.886–2.632

BR, borderline resectable; CA19-9, cancer antigen 19-9; CI, confidence interval; DSS, disease-specific survival; MTV, mean tumor volume; RFS, recurrence free survival; SD, standard deviation; SUVmax, maximum standardized uptake value; TLG, total lesion glycolysis.

*P < 0.05.

## Discussion

We evaluated the prognostic value of MTV measured on pretreatment ^18^F-FDG-PET/CT for resected patients with PDAC using two different fixed-threshold methods, the fixed-absolute threshold method and fixed-relative threshold method, with various threshold values for each method. Our study demonstrated that the fixed-absolute threshold method is superior to the fixed-relative threshold method for determining MTV and that the optimal threshold value is SUV > 3.5. This study is the first to establish the optimal segmentation method for PDAC.

Several methods for determining MTV have been proposed; however, no consensus has been established regarding the optimal method for prognostic prediction of PDAC. MTV is often determined using an approach based on two fixed-threshold methods because of its availability in clinical practice (18). In this study, we compared two types of threshold-based methods, the fixed-absolute threshold method and fixed-relative threshold method, for the prognostic prediction of PDAC.

Prognosis of PDAC may be based on tumor metabolism rather than tumor size as SUVmax and MTV show correlation with liver metastasis (**Supplementary Table 2**). The superiority of the absolute threshold method in assessing PDAC could be attributed to the fact that MTV2.5 was strongly affected by SUVmax, but MTV41% was not affected (**Supplementary Figure 1**). Furthermore, the correlation between the difference (MTV2.5–MTV41%) and SUVmax demonstrated that the relative threshold method could overestimate MTV for PDAC lesions with lower FDG uptake and underestimate MTV for lesions with higher uptake. In cases with low FDG uptake, background uptake can be misclassified into the estimated MTV, which results in overestimating the MTV with the fixed-relative threshold method (19). In lesions with intense SUVmax, the fixed-relative threshold method may define the MTV boundaries more inside the tumor than at the structural tumor boundaries. Consequently, a higher SUV in tumors could lead to underestimated MTV values. As discussed above, the fixed-relative threshold method may yield misleading results when it is applied to PDAC with a variety of sizes and signal-to background ratio on FDG-PET/CT, particularly in cases with high SUV. Patients with PDAC often underwent NAC before surgical resection, and the SUV of the tumor decreases during or after chemotherapy. The fixed-relative threshold method is not optimal to apply to such cases because the methods have high variability based on the SUVmax of the tumor. This can be problematic in predicting the prognosis of PDAC patients.

Several studies investigated the prognostic predictive utility of MTV using a fixed-absolute threshold of SUV > 2.5. We anticipated that SUV of 2.5 would also be an optimal threshold value for PDAC; however, our study revealed that SUV of 3.5 was more optimal. We speculate that MTV2.5 could not accurately reflect the tumor bulk which is responsible for the biologically malignant capability associated with metastasis. MTV2.5 might partly contain tissue volume including low-grade PDAC cells and interstitial fibrosis, which are non-high-risk factors for recurrence, and probably normal pancreatic cells. For investigating the prognostic value of MTV3.5 for recurrence types, our study revealed that MTV3.5 was a significant independent predictor for liver metastasis but not for PC recurrence and local recurrence (**Supplementary Table 2**). The volume of malignant cells with high viability (more than 3.5 of the SUV) which was meant by MTV3.5 could more strongly contribute liver metastasis and consequently affect poor prognosis. Thus, our results suggested that the threshold setting for MTV determination directly reflected the biological behavior of PDAC.

Multivariate analysis confirmed that MTV3.5 on pretreatment ^18^F-FDG-PET/CT was a better prognostic predictor in resectable PDAC than SUVmax, which was in accordance with several reports. Heterogeneity of the tumor, partial volume effect, time of SUV evaluation and body size may severely influence assessment of metabolic parameters such as SUVmax which reflect the accurate tumor characteristics. Volumetric parameters including MTV and TLG are expected to be more reliable for predicting prognosis than SUVmax because they reflect not only tumor metabolism but also whole tumor burden composed of viable malignant cells (10, 11). Although these studies reported that TLG was also an independent prognostic predictor for PDAC, the current study could not find a tendency for TLG3.5 as a predictor of PDAC. This discrepancy between our studies could be attributed to two factors. One reason may be the difference in the patients’ profiles among the cohorts. The prior cohorts included patients with resected PDAC treated with NAC, only resectable PDAC excluding borderline resectable, or advanced pancreas cancer, whereas this study included both resectable and borderline resectable PDAC without NAC. Therefore, the current results may more accurately reflect the biological behavior of resected PDAC. The second reason may be the difference in the observation periods among the cohorts. Hence, we concluded that tumor viability/metabolism (SUVmax) was an essential factor for predicting prognosis of PDAC. Volumetric information reflecting the sum of tumor viability responsible for liver metastasis as MTV3.5 could improve the accuracy of prediction for PDAC.

Currently, the treatment strategy for resectable, borderline resectable, or unresectable tumors is mainly defined by tumor configuration without considering parameters on FDG-PET/CT. Preoperative systemic chemotherapy or chemoradiation therapy may be preferred for patients with a high risk of recurrence. Additional information of tumor metabolism and tumor bulk such as MTV3.5, which is a feasible pre-surgical predictor for prognosis of patients with PDAC, may allow for a great advantage in selecting a favorable treatment strategy and could improve the prognosis of patients with PDAC. Thus, our study is of a great clinical use in establishing the effective methodology for determining volumetric parameters including MTV and TLG on pre-treatment FDG-PET/CT as potential predictors for the prognosis of PDAC patients.

This study has several limitations. First, this study was a retrospective single-center study, and the results might be subject to selection bias and thus may be biased depending on how that center managed patients with PDAC. This would impact both the values obtained and the analysis in this study. Second, the optimal threshold value of SUV 3.5 for prediction with PDAC, which we confirmed in our study, could be a specific value to our institute and not applicable to other centers. Values of SUV on FDG-PET/CT can vary depending on many technical factors: differences in machines, PET camera, image resolution, correction methods, and conditions for reconstruction (21). Further validation studies are needed to provide a normalized threshold value of SUV available for multi-center study. Third, we used only two methods, the fixed-absolute and fixed-relative threshold methods, for determining MTV on FDG PET/CT, but we did not evaluate the algorithm method. Algorithm-based methods were reported to be able to segment the tumor more accurately in tumors with wide ranges of uptake and size (18). Further investigation to explore the optimal method and compare the threshold and algorithm methods is needed. Finally, some patients showed diffuse ^18^F-FDG uptake in their pancreas parenchyma distal to the cancer, which might be associated with obstructive pancreatitis. Although we carefully avoided uptake due to pancreatitis from VOI, the measurement of MTV in those cases could be affected by the contaminated FDG uptake corresponding with pancreatitis.

Our results showed that the fixed-absolute threshold method was much more effective than the fixed-relative threshold method in determining MTV on pretreatment FDG-PET/CT for prognostic prediction in PDAC. The fixed-relative method was problematic for measuring MTV of PDAC because of the high variability according to SUVmax of the PDAC. Furthermore, we confirmed that the absolute SUV of 3.5 was the most optimal threshold value as the prognostic indicator of PDAC. MTV3.5 (cut-off value of 21.13 mm^3^) could be a powerful predictor and useful for optimizing therapeutic strategies of PDAC.

## Data Availability Statement

The raw data supporting the conclusions of this article will be made available by the authors, without undue reservation.

## Ethics Statement

The studies involving human participants were reviewed and approved by the Institutional Review Board of Tohoku University (2016-1-573). Written informed consent for participation was not required for this study in accordance with the national legislation and the institutional requirements.

## Author Contributions

YTat, CT, and KA designed the study. FM, MM, HO, and MU collected the data. YTat, CT, KA, RS, and HS analyzed the data. YTat, CT, KA, and TM reviewed the data and interpreted the statistical analysis. YTat, CT, KA, TM, and YTak drafted the manuscript. All authors contributed to the article and approved the submitted version.

## Funding

This paper was supported in part by the JST Center for Revitalization Promotion and KAKENHI Grant-in-Aid for young scientists (B) (KA18K16337). This work was supported by TUMUG Support Project (Project to Promote Gender Equality and Female Researchers) of Tohoku University (YTat) and partly supported by the Cooperative Research Project Program of Joint Usage/Research Center at the Institute of Development, Aging and Cancer, Tohoku University (KA2017-64, 2018-40, 2019-25).

## Conflict of Interest

The authors declare that the research was conducted in the absence of any commercial or financial relationships that could be construed as a potential conflict of interest.

## Abbreviations

DSS, disease-specific survival; FDG-PET/CT, fluorodeoxyglucose-positron emission tomography/computed tomography; MTV, metabolic tumor volume; NAC, neoadjuvant chemotherapy; PDAC, pancreatic ductal adenocarcinoma; RFS, recurrence-free survival; SUV, standardized uptake value; SUVmax, maximum standardized uptake value; TLG, total lesion glycolysis.
