# Supplementary material for: Defining the Optimal Method for Measuring Metabolic Tumor Volume on Preoperative 18F-Fluorodeoxyglucose-Positron Emission Tomography/Computed Tomography as a Prognostic Predictor in Patients With Pancreatic Ductal Adenocarcinoma
Source: Front Oncol. 2021 Mar 12;11:646141. doi: 10.3389/fonc.2021.646141 (PMC7994512; doi:10.3389/fonc.2021.646141)
Supplement: Supplementary file 1 [file DataSheet_1.docx]

**Supplementary Figure 1: Correlations between MTV values measured by the two different fixed-threshold methods with SUVmax and tumor size.**


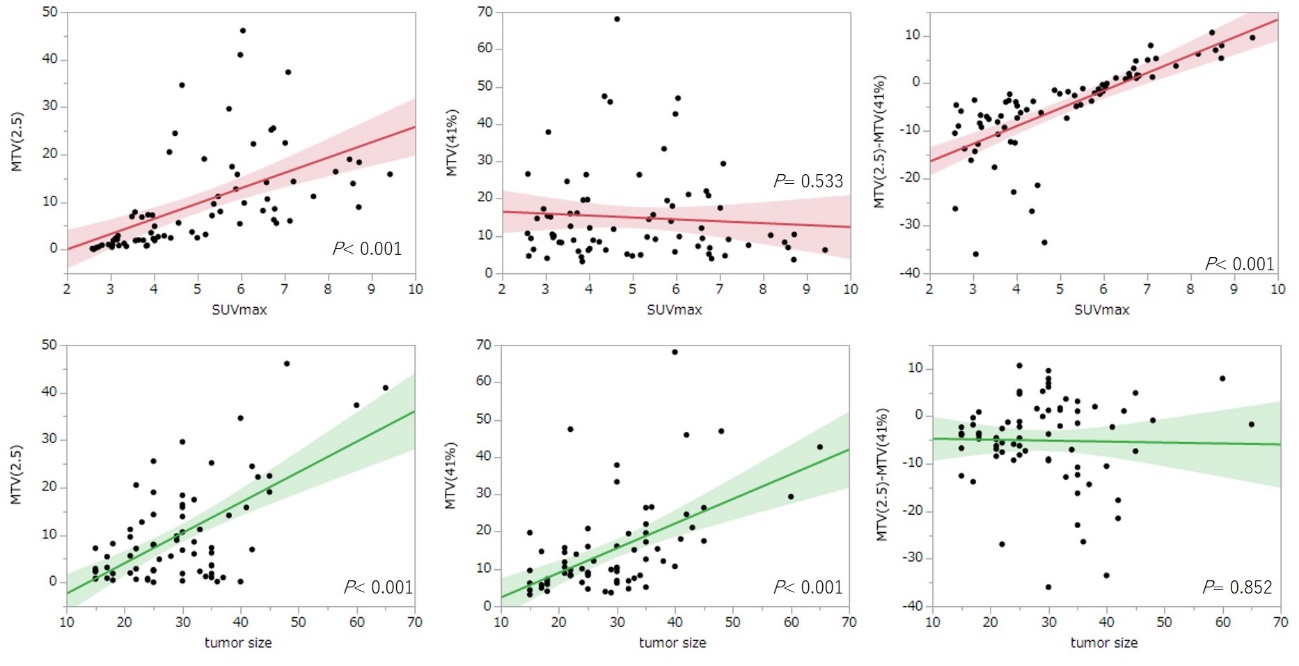
 Correlations between the representative MTV values measured by the two different fixed-threshold methods (fixed-absolute threshold method: MTV2.5; fixed-relative threshold method: MTV41%) and SUVmax and tumor size are shown (top row and bottom row, respectively). Graphs on the top right and bottom show correlations between the difference between the two MTV values and SUVmax (top) and tumor size (bottom). MTV2.5 had a strong positive correlation with SUVmax (r = 0.55, *P* < 0.001), but MTV41% had no significant correlation with SUVmax (*P* = 0.53). The difference between MTV2.5 and MTV41% showed a positive correlation with SUVmax (r = 0.72, *P* < 0.001) indicating the difference increased as the SUVmax increased. Both MTV values had significant positive correlations with tumor size (MTV2.5 vs. tumor size: r = 0.62, *P* < 0.001; MTV41% vs. tumor size: r = 0.54, *P* < 0.001). The difference between MTV2.5 and MTV41% showed no significant correlation with tumor size (*P* = 0.85).

**Supplementary Table 1:** **The area under the curves and cutoff values of MTV from the nine fixed-threshold values for 1-year DSS outcome**

|  | **MTV** | | | | **TLG** | | | |
| --- | --- | --- | --- | --- | --- | --- | --- | --- |
|  | **AUC** | **cutoff value**  **(mm^3^)** | **sensitivity** | **specificity** | **AUC** | **cutoff value**  **(mm^3^)** | **sensitivity** | **specificity** |
| Absolute SUV  >2.0  >2.5  >3.0  >3.5  % SUVmax  >35%  >40%  >42%  >45%  >50% | 0.652  0.678  0.716  0.724  0.578  0.556  0.546  0.520  0.504 | 21.13  10.63  3.59  2.46  20.01  16.69  6.57  5.08  7.25 | 0.807  0.737  0.614  0.432  0.719  0.263  0.754  0.228  0.526 | 0.500  0.563  0.750  0.750  0.438  0.875  0.375  0.875  0.563 | 0.671  0.693  0.728  0.728  0.62  0.61  0.60  0.58  0.57 | 33.05  22.74  14.45  9.99  64.15  56.91  46.64  46.76  41.06 | 0.579  0.614  0.614  0.6316  0.807  0.262  0.754  0.807  0.807 | 0.750  0.750  0.750  0.750  0.500  0.438  0.500  0.438  0.438 |

AUC, area under the curve; DSS, disease-specific survival; MTV, mean tumor volume; SUVmax, maximum standardized uptake value; TLG, total lesion glycolysis (TLG was calculated as (SUVmean) × (MTV) in each method)

**Supplementary Table 2: Multivariate analysis with MTV3.5 for recurrence types**

|  | Liver metastasis model with MTV3.5 | | | PC recurrence model with MTV3.5 | | | Local recurrence model with MTV3.5 | | | |
| --- | --- | --- | --- | --- | --- | --- | --- | --- | --- | --- |
|  | P | Hazard ratio | 95% CI | P | Hazard ratio | 95% CI | P | | Hazard ratio | 95% CI |
| Sex | 0.470 | 1.386 | 1.572–3.363 | 0.999 | <0.01 | 0 | 0.453 | 1.427 | | 0.564–3.612 |
| Age ≥66 | 0.266 | 1.804 | 0.637–5.104 | 0.687 | 1.595 | 0.164–15.523 | 0.823 | 0.902 | | 0.365–2.228 |
| CA19-9 ≥276.5 | 0.374 | 1.481 | 0.623–3.519 | 0.387 | 2.277 | 0.352–14.709 | 0.224 | 1.709 | | 0.720–4.057 |
| Resectability, BR | 0.434 | 1.428 | 0.585–3.487 | 0.345 | 2.437 | 0.384–15.469 | 0.839 | 0.907 | | 0.355–2.319 |
| MTV3.5 ≥2.46 | 0.007† | 3.649 | 1.433–9.295 | 0.139 | 5.441 | 0.578–51.291 | 0.988 | 0.993 | | 0.404–2.439 |

BR, borderline resectable; CA19-9, cancer antigen 19-9; CI*,* confidence interval; MTV, mean tumor volume; PC, peritonitis carcinomatosa*.*

†*P* < 0.01

***Evaluation of the prognostic value of MTV3.5 for recurrence types***

To determine the prognostic value of MTV3.5, we conducted Kaplan­–Meier analysis and multivariate analysis using a Cox proportional hazards regression model for recurrence types: liver metastasis, peritonitis carcinomatosa (PC) recurrence and local recurrence. On univariate analysis, the MTV3.5 cutoff value of 2.46 was strongly and significantly associated with liver metastasis (*P* = 0.002), but not with PC recurrence (*P* = 0.098) and local recurrence (*P* = 0.859). Multivariate analysis with five variables including MTV3.5, age, sex, resectability and pretreatment CA19-9 level revealed that MTV3.5 was a significant independent predictor for liver metastasis but not for PC recurrence and local recurrence.
